# IL-1β promotes ADAMTS enzyme-mediated aggrecan degradation through NF-κB in human intervertebral disc

**DOI:** 10.1186/s13018-015-0296-3

**Published:** 2015-10-06

**Authors:** Zhongyi Sun, Zhanmin Yin, Chao Liu, He Liang, Minbo Jiang, Jiwei Tian

**Affiliations:** Department of Orthopedics, School of Medicine, Shanghai General Hospital Affiliated to Shanghai Jiao Tong University, 100, Haining Road, Shanghai, 200080 China; Spine and Joint Surgery, Central Hospital of Tai An, Shandong, China

**Keywords:** IL-1β, ADAMTS enzymes, Aggrecan, NF-κB, Human intervertebral disc

## Abstract

**Background:**

The purpose of this study is to investigate IL-1β regulation of a disintegrin and metalloproteinase with thrombospondin motifs (ADAMTS-4 and ADAMTS-5) expression through nuclear factor kappa B (NF-κB) in human nucleus pulposus (NP) cells.

**Methods:**

qRT-PCR and Western blot were used to measure ADAMTS expression. Transfections and gene silencing were used to determine the role of NF-κB on cytokine-mediated ADAMTS expression and its role in aggrecan degradation.

**Results:**

IL-1β increased ADAMTS expression in NP cells. Treatment with NF-κB inhibitors abolished the inductive effect of the cytokines on ADAMTS expression. Silencing of p65 confirmed their role in IL-1β-dependent ADAMTS-4 and ADAMTS-5 expression and aggrecan degradation.

**Conclusions:**

By controlling the activation of NF-κB signaling, IL-1β modulates the expression of ADAMTS in NP cells. To our knowledge, this is the first study that shows the contribution of both ADAMTS-4 and ADAMTS-5 to aggrecan degradation in human NP cells.

## Introduction

The human intervertebral disc (IVD) is an important component of the spinal column, and its dysfunction leads to chronic low back pain (LBP) that affects 70 % of people at some point in their lives [1]. The causes of low back pain are multifactorial, although approximately 40 % of all cases involve degeneration of the intervertebral discs [2]. Intervertebral disc degeneration (IDD) is characterized by the loss of the extracellular matrix (ECM), whose major components are type II collagen (Col II) and proteoglycans (PG) [3]. More importantly, the loss of proteoglycan, predominantly aggrecan, is considered to be an early indicator of intervertebral disc degeneration and therefore a reduced ability to resist compressive load [4, 5]. Some previous studies have demonstrated that the expression and activity of matrix-degrading enzymes are increased and elicit degradation of the aggrecan in the degenerative disc [6, 7].

There are two predominant matrix-degrading enzymes that are able to hydrolyze aggrecan core proteins; these are matrix metalloproteinases (MMPs) and aggrecanases [8, 9]. There is evidence showing that there is increased expression of MMPs in the cells from degenerated IVDs [4, 10]. Although the MMPs are thought to play a role in this process [11], it has been suggested that aggrecanases may also be involved, particularly since they participate in the degradation of aggrecan in articular cartilage and osteoarthritis (OA) [12, 13].

Aggrecanase belongs to a disintegrin and metalloproteinase with thrombospondin motifs (ADAMTS) family and has the ability to degrade aggrecan. In this family, ADAMTS-4 and ADAMTS-5 are also designated as aggrecanase-1 and aggrecanase-2, respectively, because they can specifically cleave cartilage proteoglycans. Previous studies focused on osteoarthritis, which shares a similar pathogenesis with IVD degeneration, showing that there is an upregulated expression of ADAMTS in the degenerated articular cartilage [14, 15]. Unlike cartilage, in the nucleus pulposus (NP), both ADAMTS-4 and ADAMTS-5 expressions are elevated in human degenerative disc disease [8, 16]. Surprisingly, despite the importance of these ADAMTS in the pathogenesis of osteoarthritis and disc disease, only a few studies have investigated regulation of ADAMTS transcription in NP cells [17, 18], and few have used human NP cells for analysis. A clue to the mechanism lies in the finding that, in animal NP cells, nuclear factor kappa B (NF-κB) may contribute to TNF-α regulation of ADAMTS-4 and ADAMTS-5 expression [19] and that TNF-α and IL-1β also modulate ADAMTS-5 enzymatic activity through syndecan-4 [20]. Although ADAMTS have been shown to play a key role in articular cartilage and IDD, the cellular mechanisms regulating ADAMTS gene expression have, until now, not been explained. Antagonizing these proteases can potentially retard degeneration and preserve intervertebral disc tissue.

The pathogenesis of IVD degeneration is a complex process and has a number of poorly understood biological and mechanical determinants [21]. These observations beg the questions of how IL-1β controls the expression of ADAMTS-4 and ADAMTS-5 and what their relative contributions in NP cells are in terms of aggrecan degradation. In this study, we show for the first time that IL-1β controls ADAMTS-4 and ADAMTS-5 transcription in a NF-κB-dependent fashion. Furthermore, our results show that ADAMTS-4 and ADAMTS-5 are non-redundant and that both play a role in the cytokine-dependent degradation of aggrecan in human NP cells. A therapeutic strategy can conceivably target these enzymes for the structural preservation of the intervertebral disc.

## Materials and methods

### Human NP cell isolation and culture

The study was approved by the Shanghai First People’s Hospital Ethics Committee. All patients and their next of kin signed an informed consent form allowing the researchers to use IVD tissues obtained during spinal surgery. All donor patients underwent spinal fusion surgery, which requires the intervertebral disc to be resected. Patients were aged between 19 and 45 years old; mean age was 30.52 years old. Only patients aged between 18 and 45 years old were included in this study. According to the Pfirrmann grading scale [22], the discs were non-degenerative (Pfirrmann < grade III) and degenerative IVD tissue samples. All IVD tissues used in this study would have been discarded as medical waste and would not have been used in the patient’s treatment. Part of the tissues was stored in liquid nitrogen for later use; the second part was then directly sent to the cell culture laboratory super clean bench for primary culture of the nucleus pulposus cells. NP tissues were washed three times with phosphate-buffered saline (PBS; Gibco, USA), minced into small fragments, and digested in 0.25 % trypsin (Gibco, USA) and 0.2 % type II collagenase (Gibco, USA) and then placed in PBS for approximately 3 h at 37 °C in a gyratory shaker. Cells were filtered through a 70-μm mesh filter (BD, USA), and primary NP cells were cultured with growth medium (Dulbecco’s Modified Eagle’s Medium and Ham’s F-12 Nutrient Mixture [DMEM-F12; Gibco, USA], 20 % fetal bovine serum [FBS; Gibco, USA], 50 U/mL penicillin, 50 μg/mL streptomycin (Gibco USA)) in a 100-mm culture dish in a 5 % CO_2_ incubator. The cells were treated in trypsin at approximately 80 % confluence and subcultured in a 60-mm culture dish (2.5 × 10^5^ cells/well). All experiments in this study used first- or second-generation cells.

### Cell treatment

Human non-degenerative NP cells were grown to confluence in 60-mm culture dishes, underwent serum starvation overnight to synchronize the cell cycles, and were stimulated with 0~20 ng/mL recombinant human IL-1β (Peprotech, London, UK) for 24 h, and the catabolic response and NF-κB activation were observed. To justify the effectiveness of the NF-κB inhibitor, cells were pretreated with various concentrations of BAY11-7082 (Sigma-Aldrich, St. Louis, MO, USA) (2.5 μM, 5.0 μM) for 1 h, followed by treatment for 48 h with IL-1β (10 ng/mL) and BAY11-7082. Cells were then harvested for messenger ribonucleic acid (mRNA) and protein analysis.

### Immunofluorescence microscopy

The first-generation NP cells were plated in flat-bottom 24-well plates (5 × 10^3^/well) and were fixed with 4 % paraformaldehyde, permeabilized with 0.2 % Triton-X 100 in PBS for 10 min, blocked with PBS containing 5 % FBS, and incubated with antibodies against p65 (1:200) at 4 °C overnight. As a negative control, cells were reacted with isotype IgG under similar conditions. After washing, the cells were incubated with anti-rabbit secondary antibody (Jackson, USA) at a dilution of 1:200 for 1 h at room temperature. Cells were imaged using a laser scanning confocal microscope (Olympus Fluoview, Japan).

### Lentivirus infection

Lentivirus (8 × 10^8^ TU/mL) packaging of green fluorescent protein (GFP) LV-shp65 and negative control (NC) were constructed in Genechem (Shanghai, China). Cells (1 × 10^3^) were plated onto 96-well plates 48 h before the LV-shp65 and negative control were added to infected NP cells at various volumes to determine the best MOI value at which concentrations have no virus toxicity effect on cells. Ninety-six hours after infection, the GFP gene expression was observed under a fluorescence microscope, and the infected cells were collected for silencing treatment. Then, cells were harvested for protein extraction at 5 days after viral transduction. The experiment was repeated three times.

### Gene expression analysis

Total RNA was extracted from the NP cells and NP tissues by TRIzol (Invitrogen, Carlsbad, CA) according to the manufacturer’s instructions. The mRNA was analyzed with real-time polymerase chain reaction (PCR) using the ABI 7500 Fast real-time PCR system (Applied Biosystems, Carlsbad, CA). cDNA was then reverse transcribed (Thermo, USA) according to the manufacturer’s instructions. Real-time PCR reactions were done in triplicate in 96-well plates using a SYBR Premix Ex Taq Kit (TaKaRa, Dalian, China) with a final volume of 20 μL. All primers (Table 1, obtained from Sangon, Shanghai, China) were designed based on coding sequences. The cycle threshold values were obtained, and data were normalized to β-actin expression using the 2^−ΔΔCt^ method [23].

**Table 1:** Primers used for quantitative PCR

Gene	Sequence
Aggrecan	Forward: 5′TGAAACCACCTCTGCATTCCA3′
	Reverse: 5′ GACGCCTCGCCTTCTTGAA3′
P65	Forward: 5′ TGCATCCACAGTTTCCAGAAC 3′
	Reverse: 5′ CACGCTGCTCTTCTTGGAAGG 3′
ADAMTS-4	Forward: 5′ ACTGGTGGTGGCAGATGACA3′
	Reverse: 5′ TCACTGTTAGCAGGTAGCGCTTT3′
ADAMTS-5	Forward: 5′ GCTTCTATCGGGGCACAGT3′
	Reverse: 5′ CAGCAGTGGCTTTAGGGTGTAG3′
β-actin	Forward: 5′ AGCGAGCATCCCCCAAAGTT3′
	Reverse: 5′ GGGCACGAAGGCTCATCATT3′

*ADAMTS* a disintegrin and metalloprotease with thrombospondin motifs

### Western blot analysis

Nucleocytoplasmic separation and RIPA Kit were used for extraction of the nuclear protein and total protein, supplemented with phosphatase inhibitors and protease inhibitor cocktail (Biotech Well, Shanghai, China). Lysates were centrifuged for 20 min at 12,000*g*. The protein concentration was determined using the BCA protein assay (Beyotime, Jiangsu, China), and equivalent amounts of protein (40 μg) were separated by electrophoresis on sodium dodecyl sulfate polyacrylamide gel electrophoresis (SDS-PAGE) and transferred onto polyvinylidene fluoride (PVDF) membranes. After being blocked in Tris-buffered saline Tween-20 (TBS-T) with 5 % milk powder (2 h), it was then incubated with the specific antibody for NF-κB p65 (Epitomics, USA) and antibodies for ADAMTS-4, ADAMTS-5, and aggrecan (Abcom, Hong Kong, China) at 4 °C with gentle shaking overnight. After washing, membranes were incubated with the respective secondary antibody (Jackson, USA) at the appropriate concentration for 2 h at room temperature. Immunolabeling was done using enhanced chemiluminescence (ECL) reagents (Amersham Biosciences, Roosendaal, Netherlands).

### Statistical analysis

The SPSS 17.0 software (SPSS Inc., Chicago, USA) was used for statistical calculations. Student’s *t* test and analysis of variance were used for comparisons between the different groups. All experiments were repeated three times with cells from different IVD tissues, and for each experimental condition, the experiment was repeated three times; the data is represented as mean ± standard deviation. The findings were considered to be statistically significant when *P* < 0.05.

## Results

### Analysis for the expression of p65 protein in human NP tissue and NP cells

**Fig. 1:**
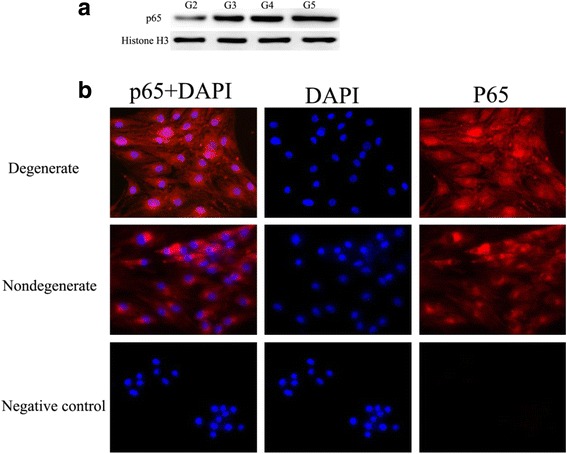
NF-κB activity was confirmed in the intervertebral disc. Part **a** shows increased levels of p65 in nuclear extracts upon degenerated disc samples compared with non-degenerated samples. Data were obtained by p65 immunoblotting of nuclear extracts (*n* = 3), and one representative sample is shown. Part **b** demonstrates that non-degenerated human NP cells show cytoplasmic localization of p65 (indicative of non-active NF-κB), while degenerated human NP cells show nuclear localization of p65 (which is indicative of active NF-κB). Data were obtained by immunocytochemistry for p65 (*n* = 2), and one representative sample is shown. Histone H3 is used as a loading control

NF-κB activity was confirmed by p65 immunoblotting of nuclear extracts (Fig. 1a): Western blots clearly showed that the p65 band in the nuclear extract of degenerated disc samples is increased compared with non-degenerated disc samples, while non-degenerated samples showed a much smaller amount of target protein. When compared to the non-degenerated group, expression of p65 protein in the degenerative group increases with an increase in the degree of degeneration of the IVD. Immunoreactivity for p65 was observed in both non-degenerated and degenerated human NP cells. In the degenerated human NP cells, the specific p65 antibody detected p65 in the nucleus (which is indicative of active NF-κB), while non-degenerated human NP cells show the cytoplasmic location of p65 (which is indicative of non-active NF-κB) (Fig. 1b).

### Gene expression of ADAMTS-4, ADAMTS-5, and aggrecan in human intervertebral discs

**Fig. 2:**
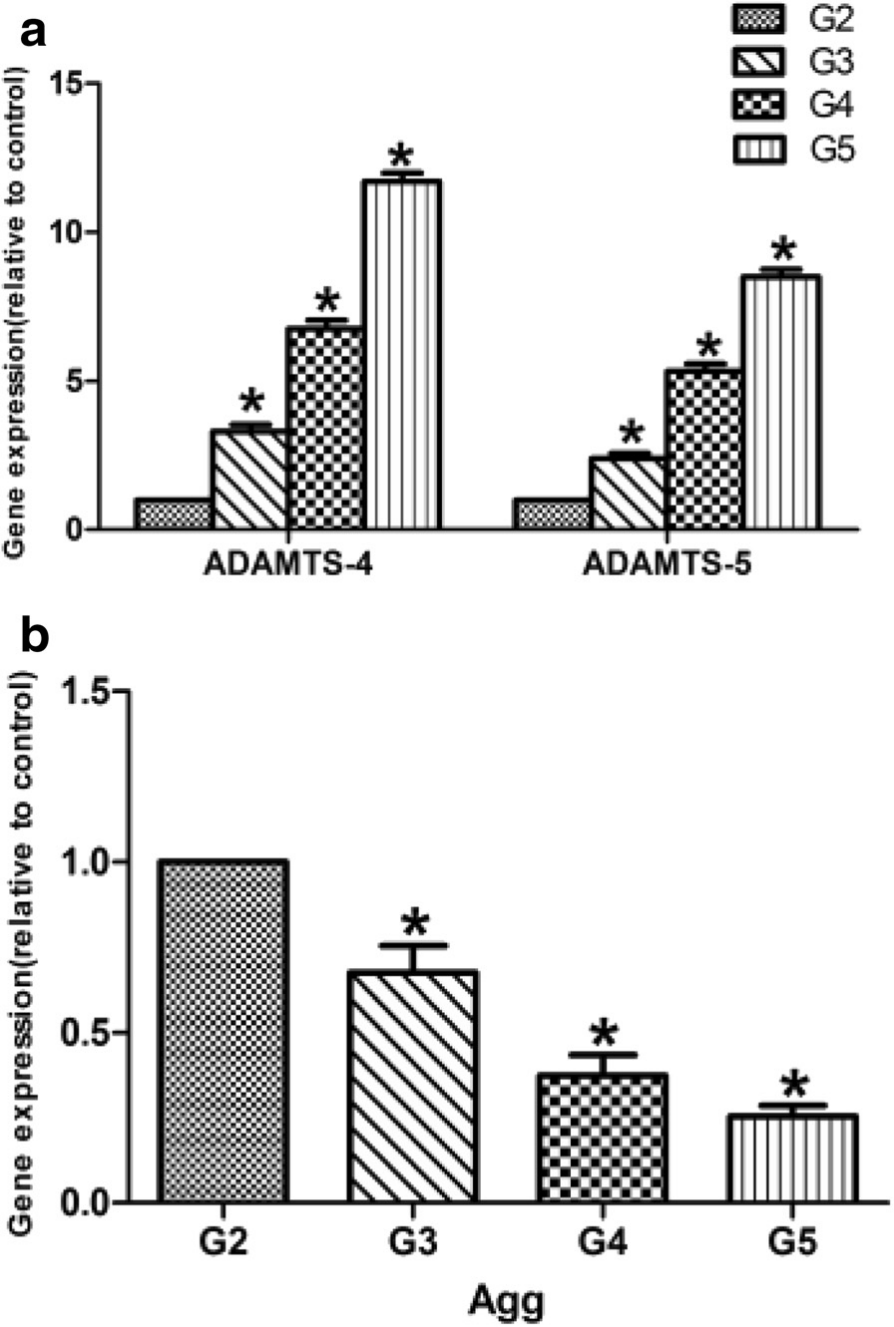
**a, b** The use of RT-PCR for the detection of ADAMTS-4, ADAMTS-5, and aggrecan mRNA expression on nucleus pulposus tissue. Values are represented as mean ± standard deviation (*n* = 6). *P* < 0.05 is considered as statistically significant. *ADAMTS* a disintegrin and metalloproteinase with thrombospondin motifs, *Agg* aggrecan, *G2* grade 2 Pfirrmann, *G3* grade 3 Pfirrmann, *G4* grade 4 Pfirrmann, *G5* grade 5 Pfirrmann. *P < 0.05 vs. control

ADAMTS-4, ADAMTS-5, and aggrecan mRNA were expressed in the nucleus pulposus of both non-degenerated and degenerated human intervertebral discs. When we compared the expression levels of the target genes, a statistically significant increase in the gene expression of ADAMTS-4 and ADAMTS-5 was seen in degenerated disc samples compared with non-degenerated disc samples (*P* < 0.05) (Fig. 2a). A corresponding decrease was seen in the aggrecan gene expression in degenerated intervertebral discs compared with non-degenerated samples (Fig. 2b).

### Expression of ADAMTS-4 and ADAMTS-5 is regulated by IL-1β in human NP cells

**Fig. 3:**
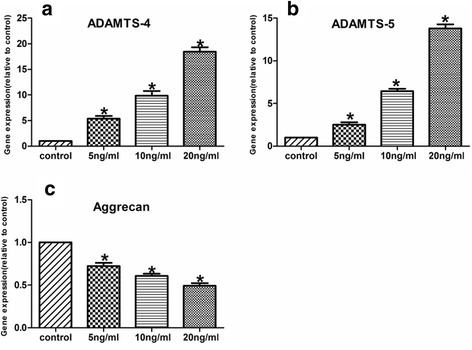
Expression and cytokine dependency of ADAMTS-4, ADAMTS-5, and aggrecan in human NP cells. **a, b** RT-PCR analysis of ADAMTS-4 and ADAMTS-5 expression by human NP cells treated with IL-1β. There was a dose-dependent increase in ADAMTS-4 and ADAMTS-5 mRNA expression by the cytokine treatment. **c** Treatment of human NP cells with IL-1β resulted in a significant decrease of aggrecan. Data are expressed as mean ± SD from six independent experiments. *P < 0.05 vs. control

**Fig. 4:**
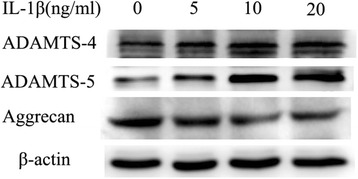
Western blot analysis of NP cells indicates increased expression of ADAMTS-4, ADAMTS-5, and aggrecan after IL-1β treatment. Data showed are representative of three independent experiments using different samples, and one representative sample is shown

The expression of ADAMTS-4 and ADAMTS-5 in human NP cells was studied using real-time PCR and Western blot analysis. To explore the premise that IL-1β concerned with disc degeneration regulates ADAMTS-4 and ADAMTS-5 expression, human NP cells were treated with IL-1β, and the expression of ADAMTS-4 and ADAMTS-5 was analyzed. Treatment with IL-1β resulted in a dose-dependent increase in ADAMTS-4 and ADAMTS-5 mRNA levels (Fig. 3a, b). In addition, we measured the level of ADAMTS-4 and ADAMTS-5 protein in a conditioned medium of treated NP cells by Western blot analysis. IL-1β treatment significantly increased ADAMTS-4 and ADAMTS-5 protein expression in human NP cells (Fig. 4). To determine if IL-1β promoted ADAMTS activity, we measured the generation of aggrecan. A significant decrease in aggrecan generation is detected when cells were treated with IL-1β compared with untreated cells (Figs. 3c and 4).

### IL-1β promotes ADAMTS-4 and ADAMTS-5 expression through activation of NF-κB signaling

**Fig. 5:**
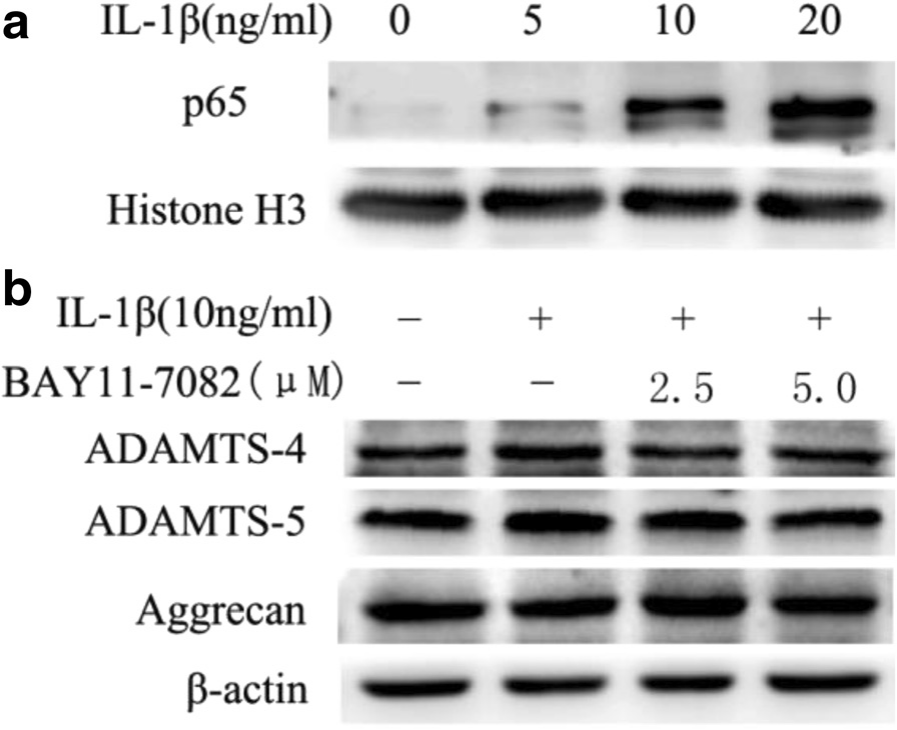
Modulation of IL-1β-dependent expression of ADAMTS-4 and ADAMTS-5 expression by NF-κB signaling in human NP cells. **a** Western blot analysis of p65 nucleoproteins after treatment of NP cells with IL-1β. **b** Western blot analysis indicates that treatment with NF-κB inhibitor completely abolished ADAMTS-4 and ADAMTS-5 protein induction by IL-1β and the level of aggrecan was significantly increased. Data are expressed as mean ± SD from three independent experiments

**Fig. 6:**
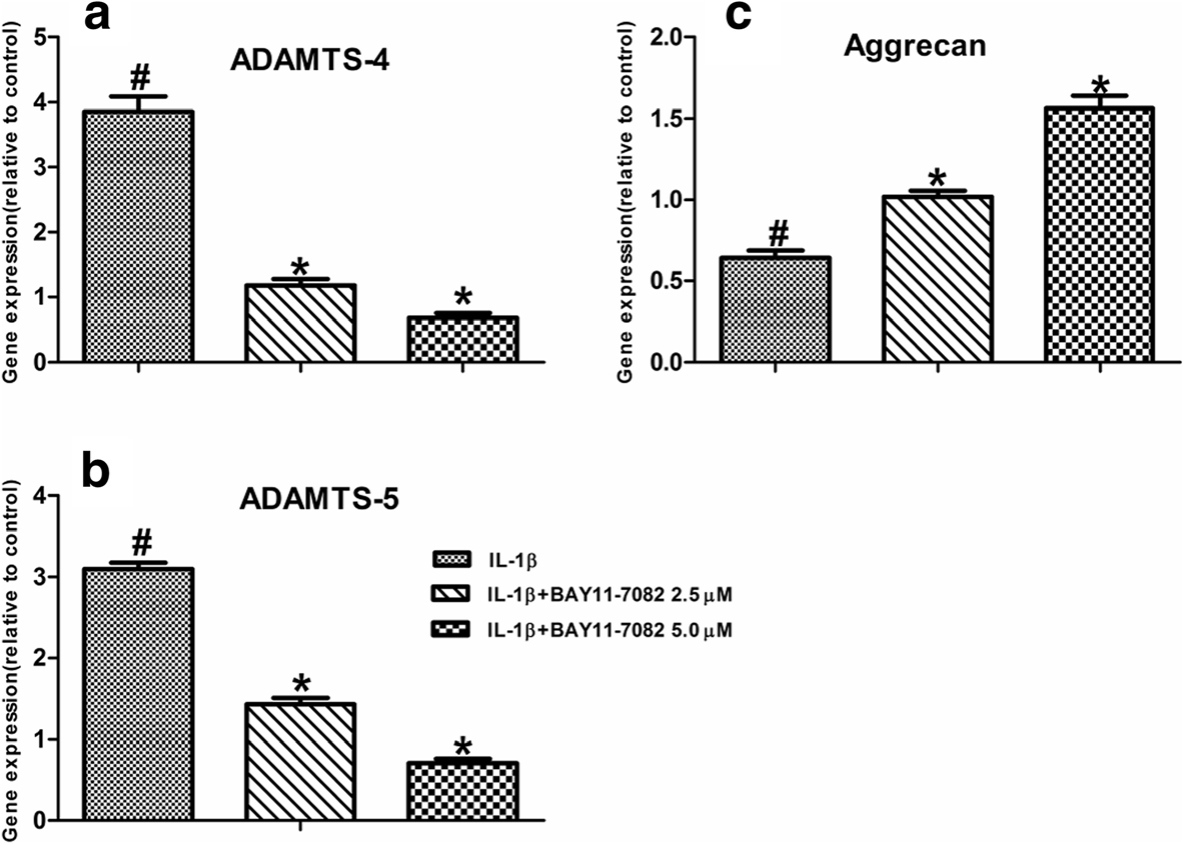
**a–c** Inhibition of NF-κB signaling resulted in a significant blocking of IL-1β-dependent induction in ADAMTS-4, ADAMTS-5, and aggrecan mRNA expression. Data are normalized with β-actin and are expressed as ratio to control cells. Control cell value = 1. Values are mean ± SD (*n* = 6 samples). *P < 0.05 vs. IL-1β alone; ^#^ *P* < 0.05 vs. control

To determine whether NF-κB signaling is required for the cytokine-dependent induction of ADAMTS-4 and ADAMTS-5 in human NP cells, we first evaluated the activation of NF-κB signaling pathways after treatment with IL-1β. After treatment with IL-1β, there was a rapid increase in p65 nucleoprotein levels (Fig. 5a). To ascertain whether the IL-1β-induced expression of ADAMTS-4 and ADAMTS-5 requires NF-κB signaling, human NP cells were pretreated with pathway-specific inhibitors. Pretreatment caused a significant suppression in the IL-1β induction of both ADAMTS-4 and ADAMTS-5 mRNA levels (Fig. 6a, b). Similarly, a pronounced decrease in the IL-1β-mediated increase in the levels of the ADAMTS-4 and ADAMTS-5 protein was seen in the presence of NF-κB pathway inhibitors (Fig. 5b). Importantly, we examined the effect of ADAMTS-4 and ADAMTS-5 expression on aggrecan degradation in human NP cells. Suppression of ADAMTS-4 and ADAMTS-5 expression resulted in a significant inhibition of IL-1β-mediated aggrecan degradation in human NP cells (Figs. 5b and 6c).

### Both ADAMTS-4 and ADAMTS-5 contribute to IL-1β-induced aggrecan degradation in human NP Cells

**Fig. 7:**
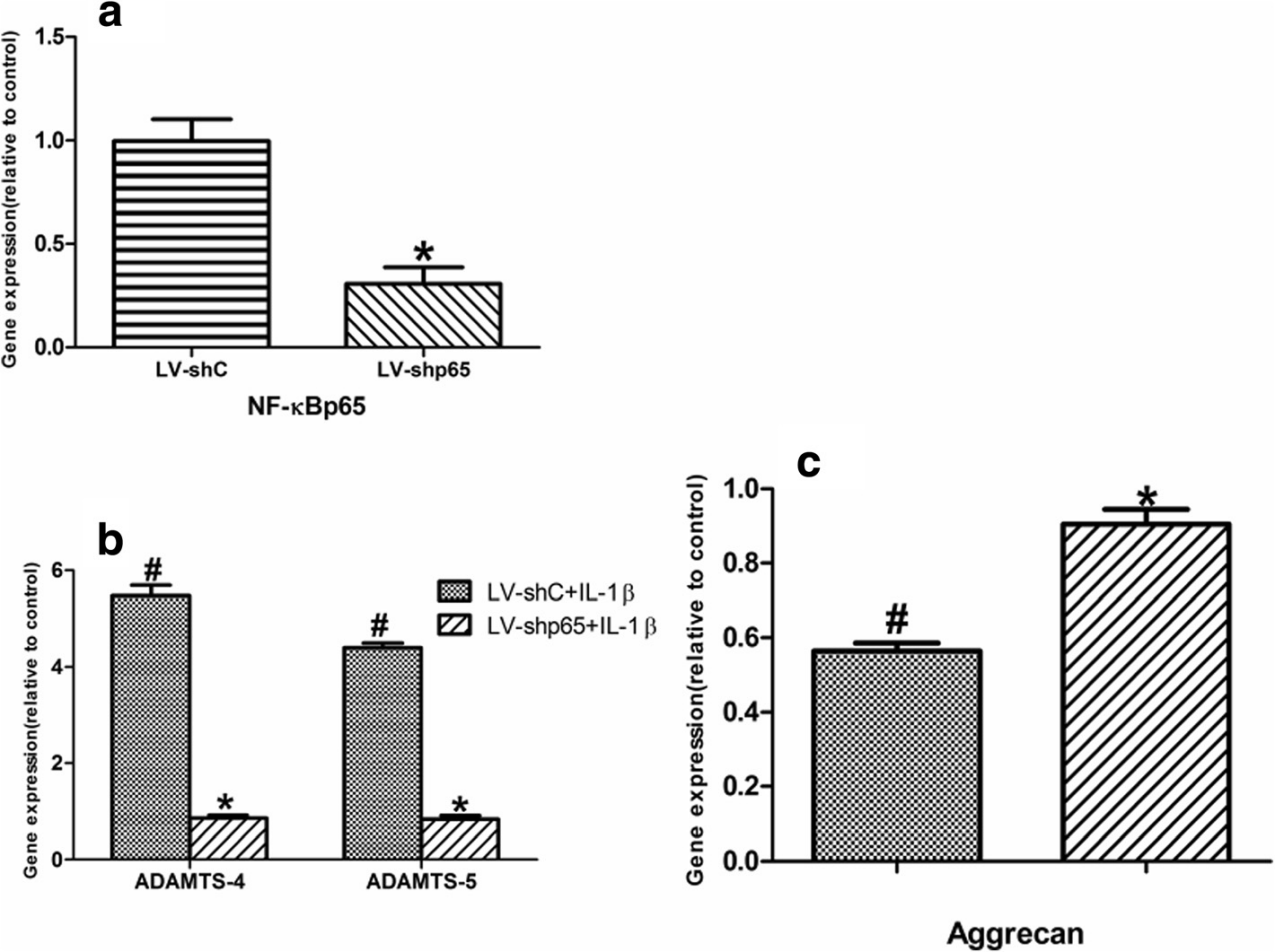
Regulation of ADAMTS-4 and ADAMTS-5 expression by NF-κB. **a** qRT-PCR analysis of cells transduced with control lentivirus LV-shC and LV-shp65. **b, c** qRT-PCR analysis of ADAMTS-4, ADAMTS-5, and aggrecan in human NP cells infected with LV-shC and LV-shp65. Note that the IL-1β-dependent increase in ADAMTS-4, ADAMTS-5, and aggrecan levels is significantly increased by suppression of the component of the NF-κB-p65 signaling pathway. Data are expressed as mean ± SD from six independent experiments. *P < 0.05 vs. IL-1β alone; ^#^ *P* < 0.05 vs. control

**Fig. 8:**
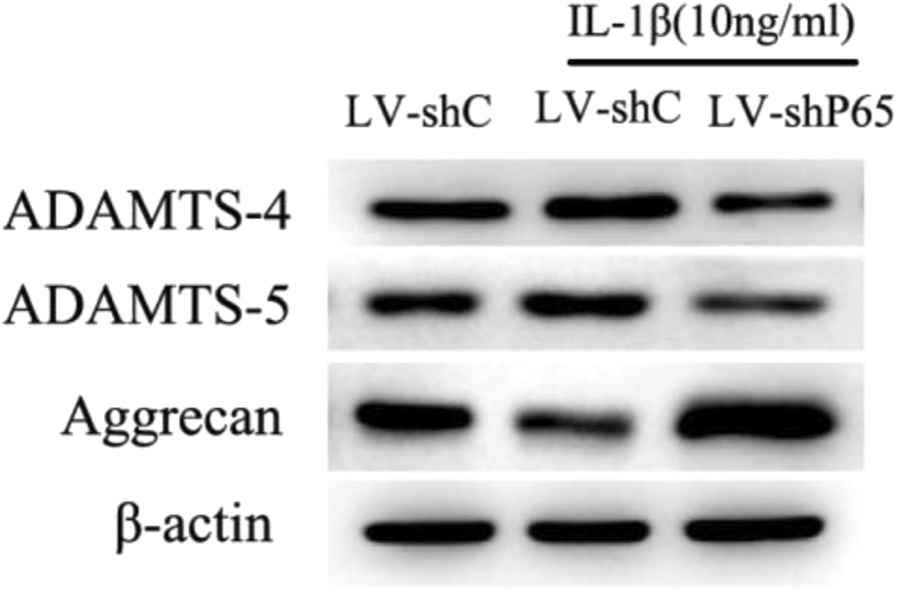
ADAMTS-4 and ADAMTS-5 promote aggrecan degradation in human NP cells. Western blot analysis of human NP cells infected with control lentivirus (LV-shC) and lentivirus expressing shRNA NF-κB-p65. Data are expressed as mean ± SD from three independent experiments

Given that IL-1β regulates ADAMTS-4 and ADAMTS-5 expression using NF-κB signaling pathways, we performed lentiviral-mediated gene silencing studies. We first silenced the expression of NF-κB p65 and then measured ADAMTS-4 and ADAMTS-5 expression in human NP cells. In human NP cells transduced with plasmid shp65, there was a significant decrease in the mRNA levels of p65, compared with cells transduced with control shRNA (Fig. 7a). Importantly, suppression of the NF-κB p65 component significantly blocked the inductive effect of IL-1β on the expression of ADAMTS-4 and ADAMTS-5 protein and mRNA levels, as well as aggrecan degradation (Fig. 7b, c and Fig. 8).

## Discussion

The experiments described in this investigation demonstrated for the first time that expression of ADAMTS-4 and ADAMTS-5, two important enzymes concerned with aggrecan degradation, is regulated by the inflammatory cytokine IL-1β through the NF-κB signaling pathways in human NP cells. A second major observation is that, by regulating ADAMTS-4 and ADAMTS-5 expression and activity, IL-1β controlled aggrecan turnover. Importantly, both ADAMTS-4 and ADAMTS-5 were required for the cytokine-dependent aggrecan degradation in human NP cells, and their function appears to be non-redundant, suggesting a possible role in intervertebral disc pathologies.

According to our gene expression studies, the results demonstrated for the first time that ADAMTS-4 and ADAMTS-5 mRNA are present in both non-degenerated and degenerated human intervertebral discs. When we compared the expression levels of the ADAMTS genes, a statistically significant increase in the gene expression of ADAMTS-4 and ADAMTS-5 was seen in degenerated disc samples compared to non-degenerated disc samples. The fact that expression is seen in non-degenerated discs could indicate a possible role for the ADAMTS enzymes in the normal turnover of aggrecan. Although ADAMTS-4 and ADAMTS-5 are considered chiefly in terms of aggrecan catabolism [24], it is involved with a number of diverse physiological functions. Moreover, the mechanism of regulation of expression is incompletely understood in human intervertebral disc. Some studies have reported that the expression of ADAMTS-4 and ADAMTS-5 is upregulated by IL-1β in chondrocytes and fibroblasts [25, 26]. In the present study, treatment of human NP cells with IL-1β clearly induced ADAMTS-4 and ADAMTS-5 expression. These results are consistent with previous reports that the inflammatory cytokines induce ADAMTS-4 and ADAMTS-5 mRNA expression in the NP [17, 20, 27]. Although the mechanism is unknown, there is some evidence to indicate that TNF-α-dependent ADAMTS-4 expression and aggrecanase activity in the NP may be regulated by NF-κB signaling [19].

The activation of the transcription factor NF-κB leads to an upregulation of proinflammatory cytokines and matrix-degrading enzymes [28–30]. We therefore investigated the NF-κB signaling activity in the human intervertebral disc. The findings of Western blot on nuclear extracts and immunofluorescence showed that the nuclear translocation of p65 correlated with degeneration. We suspected that NF-κB signaling might also be involved in the process of IVD degeneration. Indeed, chronic activation of NF-κB is associated with numerous diseases, including musculo-skeletal diseases such as osteoarthritis, osteoporosis, rheumatoid arthritis, and muscular dystrophy [31–34]. The study clearly showed that the treatment of human nucleus pulposus cells with IL-1β induced nuclear translocation of the p65 expression. We confirmed that IL-1β promoted NF-κB activation and that the signaling pathways controlled ADAMTS-4 and ADAMTS-5 expression. To further investigate whether the expression of ADAMTS-4 and ADAMTS-5 is mediated by NF-κB following IL-1β stimulation, BAY11-7082, a specific inhibitor of NF-κB, was added to the medium before IL-1β treatment. As expected, BAY11-7082 significantly reversed the effect of IL-1β on the accumulation of NF-κB and the expression of ADAMTS-4 and ADAMTS-5, therefore causing a recovery of aggrecan content in human NP cells.

In further support for the role of NF-κB in ADAMTS-4 and ADAMTS-5 regulation are loss-of-function transfection studies that measured the expression of ADAMTS-4 and ADAMTS-5. The silencing studies that demonstrated the inhibition of IL-1β-dependent ADAMTS-4 and ADAMTS-5 expression after suppression of NF-κB-p65 signaling highlight the importance of this pathway in controlling ADAMTS-4 and ADAMTS-5 expression. Moreover, the silencing of the NF-κB-p65 signaling pathway that connects ADAMTS-4 and ADAMTS-5 expression had a negative effect on aggrecan degradation in cells of the human NP. These results of the functional studies indicate that by controlling the activity of NF-κB-p65 signaling, we may modulate the expression of ADAMTS-4 and ADAMTS-5 in human NP cells. We speculate that the loss of aggrecan has been attributed to the action of the NF-κB pathway and plays a key role in IVD degradation.

## Conclusion

In conclusion, the current study suggests that NF-κB plays a major role in the pathway of the pathogenesis of IVD degeneration, leading to upregulation of ADAMTS and degradation of the disc matrix macromolecule aggrecan. We found that antagonism of NF-κB activity by genetic depletion or pharmacologic inhibition can abolish upregulation of ADAMTS-4 and ADAMTS-5 and consequently reverse the degradation of aggrecan. For the reason that inhibiting NF-κB activation may contribute to the pathogenesis of human diseases, such as disc degeneration, the suppression of NF-κB activity may represent a useful molecular target for the treatment of the NF-κB-linked human diseases.

## Abbreviations

ADAMTS: A disintegrin and metalloproteinase with thrombospondin motifs
Col II: type II collagen
ECL: enhanced chemiluminescence
ECM: extracellular matrix
IDD: intervertebral disc degeneration
IVD: human intervertebral disc
LBP: low back pain
MMPs: matrix metalloproteinases
mRNA: messenger ribonucleic acid
NF-κB: nuclear factor kappa B
NP: nucleus pulposus
OA: osteoarthritis
PG: proteoglycans
